# Unveiling the hidden diversity and functional role of Chloroflexota in full-scale wastewater treatment plants through genome-centric analyses

**DOI:** 10.1093/ismeco/ycae050

**Published:** 2024-04-11

**Authors:** Patricia Bovio-Winkler, Angela Cabezas, Claudia Etchebehere

**Affiliations:** Microbial Ecology Laboratory, Department of Microbial Biochemistry and Genomic, Biological Research Institute “Clemente Estable”, Avenida Italia 3318, 11600 Montevideo, CP, Uruguay; Departamento de sostenibilidad ambiental, Instituto Tecnológico Regional Centro Sur, Universidad Tecnológica, Francisco Antonio Maciel s/n, 97000, Durazno, CPUruguay; Microbial Ecology Laboratory, Department of Microbial Biochemistry and Genomic, Biological Research Institute “Clemente Estable”, Avenida Italia 3318, 11600 Montevideo, CP, Uruguay

**Keywords:** Chloroflexota, methanogenic reactors, activated sludge, metagenome assembled genomes, meta-analysis

## Abstract

The phylum Chloroflexota has been found to exhibit high abundance in the microbial communities from wastewater treatment plants (WWTPs) in both aerobic and anaerobic systems. However, its metabolic role has not been fully explored due to the lack of cultured isolates. To address this gap, we use publicly available metagenome datasets from both activated sludge (AS) and methanogenic (MET) full-scale wastewater treatment reactors to assembled genomes. Using this strategy, 264 dereplicated, medium- and high-quality metagenome-assembled genomes (MAGs) classified within Chloroflexota were obtained. Taxonomic classification revealed that AS and MET reactors harbored distinct Chloroflexota families. Nonetheless, the majority of the annotated MAGs (166 MAGs with >85% completeness and < 5% contamination) shared most of the metabolic potential features, including the ability to degrade simple sugars and complex polysaccharides, fatty acids and amino acids, as well as perform fermentation of different products. While Chloroflexota MAGs from MET reactors showed the potential for strict fermentation, MAGs from AS harbored the potential for facultatively aerobic metabolism. Metabolic reconstruction of Chloroflexota members from AS unveiled their versatile metabolism and suggested a primary role in hydrolysis, carbon removal and involvement in nitrogen cycling, thus establishing them as fundamental components of the ecosystem. Microbial reference genomes are essential resources for understanding the potential functional role of uncultured organisms in WWTPs. Our study provides a comprehensive genome catalog of Chloroflexota for future analyses aimed at elucidating their role in these ecosystems.

## Introduction

Wastewater treatment systems are artificial ecosystems in which a microbial consortium degrades organic matter with the aim of cleaning the water. In this way, water with sufficient quality is obtained to be discharged into water courses without causing major environmental problems. The most conventional systems are activated sludge systems, which consist of an aerated reactor in which aerobic microorganisms degrade the organic matter [1]. For wastewater with a high organic matter concentration or for solid waste, it is more convenient to use anaerobic systems in which the organic matter is converted into methane by the action of a consortium of bacteria and archaea [2]. The advantage of anaerobic systems is that methane is obtained that can be used as fuel. Both systems are widely used on full scale wastewater treatment plants (WWTPs) and can be considered established technologies [1, 3]. However, the microbiology of these complex systems still presents several knowledge gaps. Despite the great difference between the two systems (aerobic and anaerobic) the phylum Chloroflexota have been detected in high abundance in the microbial communities from both methanogenic [4, 5] and activated sludge systems [6, 7]. Previous studies have indicated that Chloroflexota contributes to the formation of the filamentous matrix around which flocs and granules are formed [8, 9]. However, several authors have associated the overgrowth of some genera of Chloroflexota with bulking episodes and poor sludge–water separation, mainly in activated sludge systems but also in full-scale methanogenic reactors and lab-scale anammox reactors [10–14]. In activated sludge reactors, the biomass is flocculent in nature [15, 16]. Meanwhile, in granular sludge-based bioreactors, microbial biomass grows in the form of granules, which are small, self-immobilized, spherical, denser and more compact than activated sludge flocs [17]. In contrast, in anaerobic digester systems, the microbial biomass typically does not exhibit granulation but is instead dispersed [18, 19]. Bulking episodes occur when a significant portion of filaments extend beyond the confines of granules and flocs, protruding into the bulk water. In a recent study, most of the Chloroflexota members in activated sludge systems formed thin and short trichomes integrated into the floc structure, which are unlikely to form the typical inter-floc bridging that hinders the settling of activated sludge flocs [20]. To understand under which conditions certain Chloroflexota members overgrow, it is necessary to isolate them in pure culture. However, despite reaching relative abundances of up to 35% in these ecosystems [21], Chloroflexota has long been considered a group of the yet-to-be-cultured microbes that are recalcitrant to cultivation and isolation [10, 22]. The most widely accepted hypothesis is that members of the Chloroflexota phylum are notoriously difficult to cultivate due to slow growth, particularly those belonging to the Anaerolineae class [23, 24]. Consequently, they are easily outcompeted by fast-growing heterotrophic bacteria.

Advancements in metagenome data analysis have made it possible to assemble genome sequences, providing access to detailed information about the metabolic potentials of these organisms [21, 25, 26]. Based on the physiology of isolates, assembled genomes from metagenomes and in situ characterization [20, 26], Chloroflexota members have been proposed to primarily function as heterotrophic and facultative anaerobic bacteria [20]. For instance, they are capable of hydrolyzing complex organic matter, fermenting carbohydrates and amino acids, and degrading cellular debris [4, 7, 8, 22, 26–32]. It has also been suggested that Chloroflexota members may play a role in the nitrogen cycle, thereby improving the nitrogen removal performance in anammox bioreactors [33, 34] and activated sludge systems [20, 21].

Due to the lack of pure cultures, microbial reference genomes are essential resources for expanding the phylogenomic representation of Chloroflexota and comprehend their functional role in WWTPs. This has direct implications for reactor performance, particularly in preventing and controlling bulking problems caused by their overgrowth.

In our previous investigations, we studied the diversity of Chloroflexota by analyzing amplicon sequencing data obtained from 62 full-scale methanogenic reactors. Then, we explored the potential metabolic role of 17 Chloroflexota members through genome-centric metagenomic analyses using samples collected from a methanogenic reactor, an activated sludge reactor and anammox reactor [5, 21, 35]. The outcomes of these studies present exciting prospects for addressing questions related to the taxonomic composition and potential metabolic functions of Chloroflexota members. This is accomplished with the added robustness of determining these aspects through a meta-analysis of metagenome-assembled genomes (MAGs).

In this study, we conducted an analysis of 264 medium- and high-quality metagenome-assembled genomes of Chloroflexota, retrieved from metagenomic public data from activated sludge and methanogenic full-scale reactor. The objective of our research was to address the following questions: Is the Chloroflexota taxonomic composition the same in activated sludge and methanogenic reactors? Does the metabolic potential of Chloroflexota differ between both systems? Which carbon compound degradation pathways do they have?

## Materials and methods

Collection of public metagenomic data and metagenome-assembled genomes.

This meta-analysis included sequences from 18 published papers and data from the present work, which used shotgun sequencing to survey the microbial community in full-scale activated sludge (AS) and methanogenic (MET) reactors. We compiled the data from 87 full-scale reactors distributed in 15 countries, comprising 45 AS and 42 MET reactors (36 digesters and 6 upflow anaerobic sludge blanket (UASB) reactors) (Table 1, Supplementary Data 1). For the analysis, digesters and UASB type reactors were grouped into MET reactors.

**Table 1:** Summary of the data analyzed indicating, reactor type, country, number of reactors and the reference.

**Reactor type**	**Country**	**Reactors**	**Reference**
Aerobic activated sludge reactors (45 reactors)	Uruguay	1	This work
	Uruguay	1	Bovio-Winkler et al. 2023 [5]
	Singapore	1	Haryono et al. 2021 [52]
	China	1	Liang et al. 2021^a^ [42]
	Argentina	1	Pérez et al. 2019 [53]
	Germany	2	Schneider et al. 2021 [54]
	Denmark	23	Singleton et al. 2021 [55]
	Hong Kong	1	Wang et al. 2021 [56]
	Denmark	1	Ye et al. 2020 [57]
	China	8	
	USA	1	
	Argentina	1	
	Slovenia	1	
	Singapore	1	
	Taiwan	1	Yang et al. 2020^a^ [45]
Methanogenic digesters (36 reactors)	Sweden	4	Brandt et al. 2020 [58]
	Germany	2	
	Denmark	1	Campanaro et al. 2020 [59]
	Sweden	1	
	Spain	2	
	Denmark	2	Hao et al. 2020^a^ [60]
	China	2	Li et al. 2022^a^ [41]
	China	6	Ma et al. 2021 [61]
	France	1	Puig-Castellví et al. 2021^a^ [43]
	UK	8	Raguideau et al. 2021 [62]
	China	5	Fan et al. 2022^a^ [46]
	Germany	2	Schneider et al. 2021 [54]
Methanogenic UASB type reactor (6 reactors)	Uruguay	1	This work
	Uruguay	1	Bovio-Winkler et al. 2023 [5]
	China	2	Liang et al. 2021^a^ [42]
	Japan	1	Park et al. 2020^a^ [63]
	Taiwan	1	Yang et al. 2020^a^ [45]

^a^Indicates in which works we performs the assembly and binning.

Metagenome-assembled genomes (MAGs) from 11 studies were available in the National Center for Biotechnology Information (NCBI), Sequence Read Archive (SRA), and/or European Nucleotide Archive (ENA) databases. Metagenomic assembly and contig binning were conducted for two reactors from the current study and 16 reactors from seven additional studies (Table 1, Supplementary Data 1) [21, 36–42] since MAGs had not been previously assembled or were not available in public databases. All shotgun sequencing datasets were generated using the Illumina platform with paired-end sequencing strategy. For samples taken from both reactors in the present study we followed the protocols for sampling, DNA extraction and metagenome sequencing of samples described in a previous publication [21]. Briefly, total DNA was extracted from samples from two full-scale reactors located in Uruguay: a full-scale Internal Circulation (IC) methanogenic reactor treating effluent from a brewery (FNC) and a full-scale activated sludge reactor treating oil and grease (CO). For DNA extraction, sludge samples were thawed and centrifuged (5 min, 10 000 g). Approximately 0.35 g of wet pellet was used for DNA extraction with the ZR Soil Microbe DNA MiniPrepTM kit (Zymo Research) according to the manufacturer’s instructions. The quality of the extracted genomic DNA was determined by 1% agarose gel electrophoresis (Nucleic Acid Stain, GoodViewTM, Beijing) and stored at −20°C until further use.

### Metagenomic assembly and contigs binning

Default parameters were used with all software, unless otherwise specified. The overall quality of metagenomes reads from the present work and for the seven studies was assessed using FastQC (v0.11.8) [43]. Basic statistics of shotgun sequence statistics (number of reads, read length, dataset size) of all metagenomes used for assembly in this study are provided in Table S1. Reads were then trimmed using Trimmomatic (v0.39) [44] to remove adapters and bases below a quality score of 25 (HEADCROP:10, LEADING:3, TRAILING:3, SLIDINGWINDOW:4:24, MINLEN:75). For each work, clean metagenomic reads were assembled into contigs using MEGAHIT v1.2.9 [45] (—k-min 21, —k-max 123, —k-step 4, —min-contig-length 1000). The contigs were then binned (—maxbin2, —concoct, —metabat2) and refined (−c 50, −x 10) using metaWRAP v1.3.2 [46]. To increase the completion of the bins, and reduce contamination, metaWRAP reassemble_bins module (−c 50 -x 10 options) was used. The resulting MAGs were analyzed along MAGs from the other 11 studies (Table 1). All MAGs were identified using GTDB-Tk v2.3.0 [47] and the Genome Taxonomy Database (GTDB) R214 [48] (Supplementary Data 1). The sequence statistics of the genomes were estimated using QUAST [49]. The completeness and contamination were estimated using CheckM2 (v1.0.1) [50].

To ensure stricter genome quality control, we selected Chloroflexota MAGs (retrieved from databases and assembled in the present work) with a completeness over 50%, a contamination level of less than 5%, a genome quality score (QS) greater than 50 (defined as the estimated completeness of a genome minus five times its estimated contamination) and free of chimerism as determined by GUNC [51]. We used a CSS of 0 for all genomes, a CSS closer to a value of 0 indicates that a genome is free of contamination and all genes are assigned to the same taxonomy, whereas a CSS score closer to 1 indicates chimerism. As a result, 522 high- and medium-quality MAGs were retained (MAGs_QS_). From this set of MAGs obtained, we selected for diversity and metabolism analysis (see further details below) the highest number of MAGs possible for each case. For diversity and phylogenomic analysis, we used dereplicated MAGs (completeness and redundancy score equal to or greater than 50). Meanwhile, for metabolism analysis, dereplicated MAGs with a completeness greater than 85% were utilized, as there is evidence suggesting that the completeness of genomes significantly influences the recovered functional signal [64]. We dereplicated the MAGs_QS_ at 95% average nucleotide identity (ANI) using dRep v3.2.2 [65] with the parameters -comp 50 -con 10 -sa 0.95 to identify the representative species in the genome tree (where ANI refers to alignment of at least 95% similarity for any section of the genome spanning a minimum of 10% of the bin length). These MAGs_dRep_ were used for phylogenomic and taxonomic analyses. The 16S rRNA gene sequences of Chloroflexota MAGs_dRep_ were identified using barrnap v0.993 (https://github.com/tseemann/barrnap).

### Phylogenomic analysis

To determine the phylogenetic position of the 264 MAGs_dRep_ (and 96 reference genomes retrieved from NCBI in February 2021) a phylogenomic tree based on concatenated alignments of 120 single copy marker genes was constructed using FastTree v2.1.11 (JTT, SH support values) applying the JTT protein substitution model for tree inference. From 264 MAGs_dRep_, 16 MAGs were renamed according to [20]. Newick format tree file was uploaded to iTOL v6, a web-based tool for annotating and editing trees [66].

### Gene annotation

Gene annotation was performed using MAGs_dRep_ with more than 85% completeness and less than 5% contamination resulting in 166 MAGs (MAG_sannot,_ 102 MAGs for AS and 64 MAGs for MET reactors). The annotation was carried out with the “annotate” function of EnrichM v0.6.5 (https://github. com/geronimp/enrichM), using the Kyoto Encyclopedia of Genes and Genomes (KEGG) Orthologies (KOs) [67] and Carbohydrate Active enzyme (CAZy) database [68]. EnrichM’s “classify” function was used to calculate the completeness of KEGG modules. “Modules” are groups of genes organized by steps in a metabolic pathway as defined by KEGG. Only KEGG modules with 100% completeness in at least one MAG were kept in the downstream analyses.

### Statistical analysis and data visualization

Statistical analyses and graphs were performed in R [69] using the following packages: vegan [70], ampvis2 [71], ggplot2 [72] and pheatmap [73]. Shapiro–Wilk’s test was used to determine whether the estimated genome size (Mb) and coverage were normally distributed. Since the estimated genome size was not normally distributed, a Kruskal-Wallis test (used to test equality of means when data is not normally distributed) was used to compare the estimated genome size (Mb) between AS and MET reactors. To determine significant differences in the coverage of Chloroflexota between AS and MET reactors, the Mann–Whitney U test (to test equality of means, Bonferroni corrected) were applied using Past (v3.21) [74]. Hierarchical cluster analysis (Euclidean distance) was used to cluster the CAZy families and KEGG pathways.

## Results and discussion

### Chloroflexota metagenome assembled-genomes dataset

**Figure 1:**
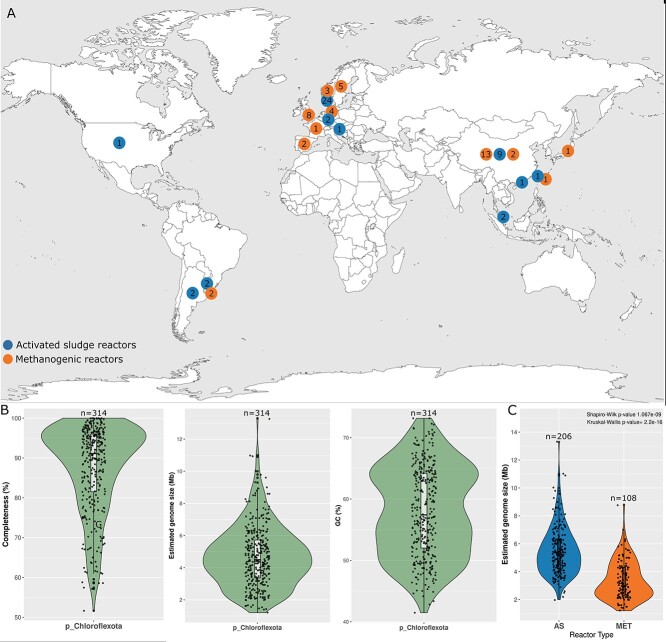
A) Geographic distribution of the 87 reactors. The numbers inside the circles indicate the number of reactors in each country. B) Violin plots showing the estimated completeness (%), estimated genome size (Mb) and GC (%) content for the representative species of Chloroflexota (MAGs_dRep_ = 264) obtained from all WWTPs. C) Violin plots showing the comparison of the estimated genome size (Mb) between MAGs_dRep_ from AS and MET reactors (Shapiro–Wilk normality test <0.05, non-parametric test Kruskal–Wallis *P*-value <0.05).

To explore the diversity and potential metabolic role of the phylum Chloroflexota in full-scale WWTPs, we compiled publicly available data from 18 studies comprising 87 reactors distributed in 15 countries (Fig. 1A, Table 1, Supplementary Data 1). The reactors were divided into AS (45 reactors) and MET (42 reactors) (Table 1). After applying the quality score of >50 and dereplication, we retained 264 MAGs_dRep_ (206 for AS and 108 for MET) (Supplementary Data 1). MAGs_dRep_ were estimated to be 91.1% (median) complete, 2.8% (median) contaminated, with an estimated genome size of 4.5 Mb (median) and a GC content of 57.4% (Fig. 1b). The estimated genome size (Mb) in MAGs_dRep_ from AS was significantly larger than in MET reactors (Kruskal–Wallis *P*-value <0.05) (Fig. 1C). Some ecological factors are correlated with genome size [75]. For example, oxygen is known to promote larger genome sizes [76]; there is a negative correlation between genome size and temperature [77, 78] and species with larger genome-sizes may dominate environments where resources are scarce but diverse [79–82]. Activated sludge reactors are oxygen-rich ecosystems, most of them treat sewage containing variable compounds and operational temperatures are lower than in digesters (Supplementary Data 1). For that reason, species from Chloroflexota in these environments may possess a more diverse set of genes and larger genome sizes. In contrast, MET reactors, which operate in anaerobic conditions, have higher temperatures and treat substrates such as sludge, manure, and other materials with less variability and high concentration of organic matter [83]. These conditions favor the presence of small genomes.

### Different Chloroflexota communities dominate aerobic and methanogenic reactors

**Figure 2:**
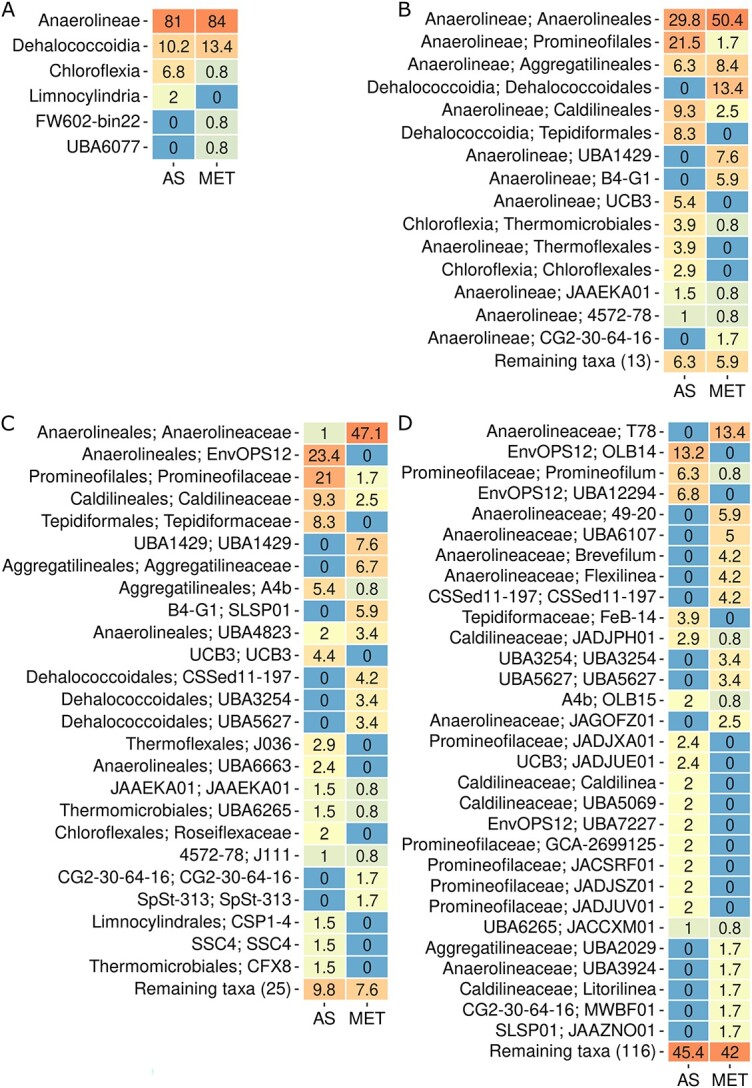
Heatmap showing the relative abundance of the Chloroflexota MAGs_dRep_ in activated sludge (AS) and methanogenic reactors (MET) classification at A) class level, B) order level, C) family level and D) genus level.

**Figure 3:**
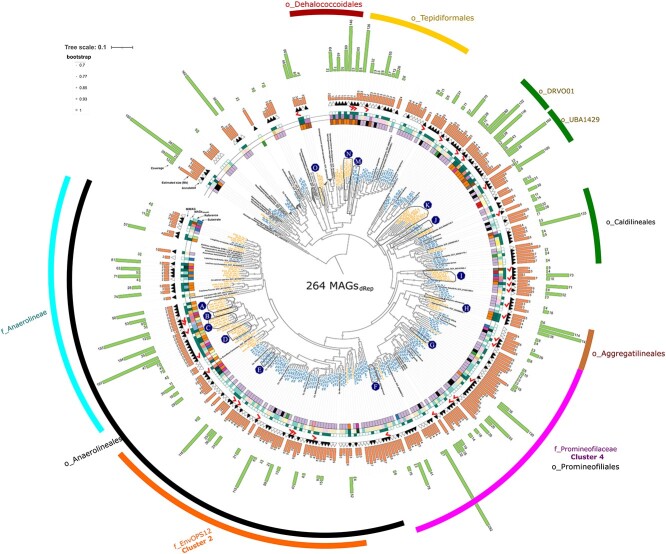
Phylogenomic tree of Chloroflexota MAGs_dRep_ (n = 264) and reference genomes retrieved from NCBI (n = 96). Letters from A to O indicate clusters containing high-quality (HQ) draft MAGs with no representative cultures. Genomes from the phylum Thermotogota were used as outgroup to root the tree.

The Chloroflexota community taxonomic composition determined using MAGs_dRep_ (n = 264) revealed that the class Anaerolineae represented 81% and 84% of these MAGs_dRep_ in AS and MET reactors, respectively (Fig. 2A). These results were in accordance with earlier reports in WWTPs [5, 84]. Within Anaerolineae class, MAGs_dRep_ belonging to Anaerolineales (29.8%), Promineofilales (21.5%) and Caldilineales (9.3%) orders were the most abundant in AS reactors (Fig. 2B). Meanwhile, Anaerolineales (50.4%) and Aggregatilineales (8.4%) were the most abundant orders in MET reactors. Dehalococcoidia class was the second most abundant class in AS (10.2%) and MET (13.4%) reactors (Fig. 2a). Within Dehalococcoidia class, Tipidiformales was only present in AS reactors while Dehalococcoidales only in MET reactors. Coverage values were collected for 54% of the MAGs_QS_ (coverage was used as a proxy for the relative abundance of MAGs in complex communities), as this is a value frequently reported for MAGs in most studies (Supplementary Data 1), unlike relative abundance. The MAGs_QS_ from MET showed higher coverage than those from AS reactors (normal distribution Shapiro–Wilk *P*-value = 0.00049, Mann–Whitney U test (Bonferroni corrected) *P*-value = 0.0004924), indicating a greater abundance of Chloroflexota in methanogenic systems (Fig. S1) There are no reports comparing the abundance of Chloroflexota genomes between AS and MET reactors, thus our results would be of interest from both ecological and process perspectives. In our previous study, amplicon sequencing results showed that methanogenic reactors presented a higher relative abundance of Chloroflexota than activated sludge reactors [21]. Most of the families and genera of Chloroflexota were not shared between AS and MET reactors (Fig. 2C and D). These results were clearly depicted in the phylogenomic tree constructed using MAGs_dRep_ (Fig. 3), where distinct clusters at the family level were formed based on AS or MET reactors. In a recent work by Petriglieri [20], 29 newly described species of Chloroflexota were designated as *Candidatus*. We incorporated 21 of these species covering all Chloroflexota genera studied in this work into our dataset and phylogenomic tree. Additionally, we recovered 16 MAGs that met the criteria for high-quality (HQ) draft MAGs (Supplementary Table 2), in accordance with minimum information about a MAG (MIMAG) standard [85] (MAGs_MIMAG_). These MAGs_MIMAG_ are within clusters without representative species (isolates or Candidatus membes) (Fig. 3 clusters A-O), and were not classified at the genus level (3 MAGs) or species level (16 MAGs) using the GTDBtk database. This suggest that they are new species, highlighting the importance of the genomics analyses of Chloroflexota. Microbial reference genomes are essential resources for understanding the functional role of specific organisms in the different ecosystems. However, according to our results, an estimated 68% of Chloroflexota species analyzed in the present work lack a reference genome (Supplementary Data 1).

### Potential role of Chloroflexota as hydrolytic and heterotrophic bacteria

**Figure 4:**
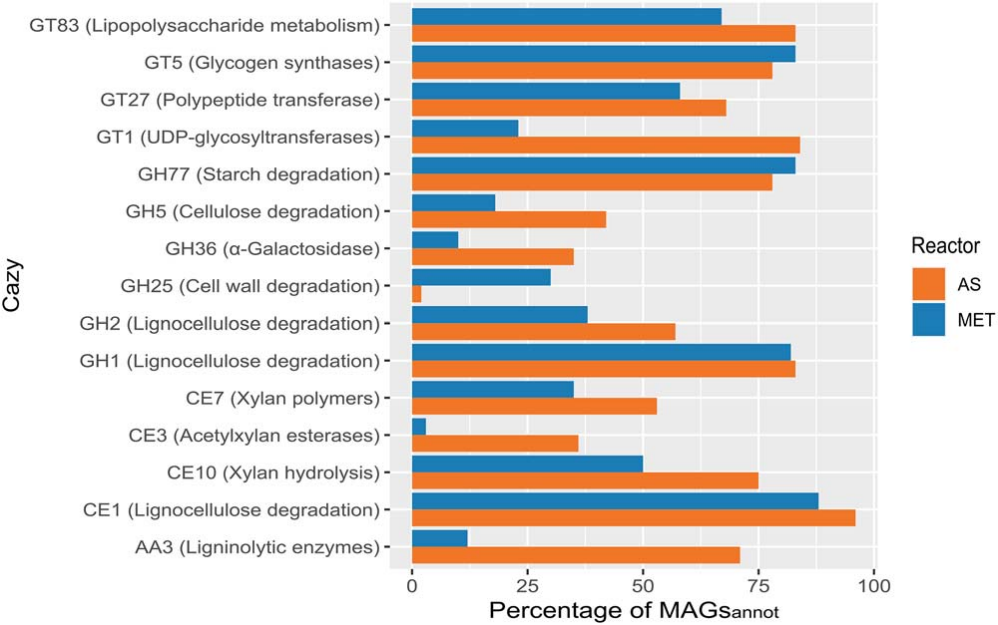
Analysis of the CAZy enzymes in Chloroflexota MAGs from AS and MET reactors. CAZy families found in more than 50% of the MAGs_annot_ (annotated using the dbCAN2 carbohydrate-active enzyme (CAZy) domain HMM database). Glycoside hydrolase (GH), Glycosyl transferase (GT), carbohydrate esterase (CE), auxiliary activities (AA). Percentages of MAGs containing a Cazy family were calculated relative to the total number of MAGs in each reactor type (AS reactor vs MET reactors).

To confirm the potential capacity of Chloroflexota to recycle soluble microbial products by acting as hydrolytic bacteria, we performed the annotation of MAGs_annot_ (166 MAGs) using Carbohydrate-active Enzymes (CAZy) database. Overall, all MAGs_annot_ were enriched on average in 8.6 classes (ranging from 0 to 25 of 1414) of glycoside hydrolases (GH), 4.9 classes (ranging from 0 to 11 of 801) of glycosyltransferases (GT) and, 3 classes (ranging from 0 to 6 of 487) of carbohydrate esterases (CE) (Fig. S2). This evidence suggests that polysaccharide degradation may represent the most widespread functional activities in the phylum Chloroflexota (Figs 4 and S3, Supplementary Data 2). These CAZy enzymes are capable of breaking down various substrates, including starch (GH77), lignocellulose (GH1, GH2, CE1), xylan polymers (CE7), lignin (AA3) and glycogen (endoglucanase and beta-glucosidase, blgX and/or blgB).

The differences in classes of CAZy enzymes between MAGs_AS_ and MAGs_MET_ were limited (MAGs from activated sludge reactors: MAGs_AS_; MAGs from methanogenic reactors: MAGs_MET_). Among MAGs_AS_, AA3 (ligninolytic enzymes), GH5 (endo-type cellulases), GH36 (α-Galactosidase), and CE3 (acetylxylan esterases) were more prevalent compared to MAGs_MET_ (Fig. 4). In contrast, MAGs_MET_ showed a higher prevalence of GH25, which is associated with cell wall degradation, such as lysozyme.

The presence of cellulolytic enzymes in genomes from MET reactors (fed with primary sludge from AS reactors) and AS reactors (treating municipal wastewater) can be attributed to their high cellulose content, which originates mainly from toilet paper and constitutes about 35% of the suspended solids in the influent [86, 87]. Furthermore, in our set of MAGs_annot_, several MET reactors treated plant material. The cellulolytic potential of Chloroflexota was previously reported in Ktedonobacteria lineage (presence of acetylxylan esterases belonging to the CE1) [88] and some Anaerolineae members (several GHs families) [89].

The common ability of Chloroflexota members to grow on starch (alpha-glucan polysaccharides) [30, 31] and cellulose [90] is important considering the bottlenecking polysaccharide hydrolysis step of anaerobic digestion. This is in accordance with previous in situ studies, which revealed high levels of surface associated hydrolytic enzymes and their involvement in the breakdown of complex organic compounds [8, 91].

The diverse repertoire of CAZyme genes provides the basis for a flexible carbohydrate metabolism within the microbial community [86]. Under carbon-deficient conditions that prevail in nutrient removal WWTPs, carbon and energy sources supporting further growth of Chloroflexota members may originate from sugars released from the hydrolysis of cellulose, exopolysaccharides and cellular detritus [20, 26].

**Figure 5:**
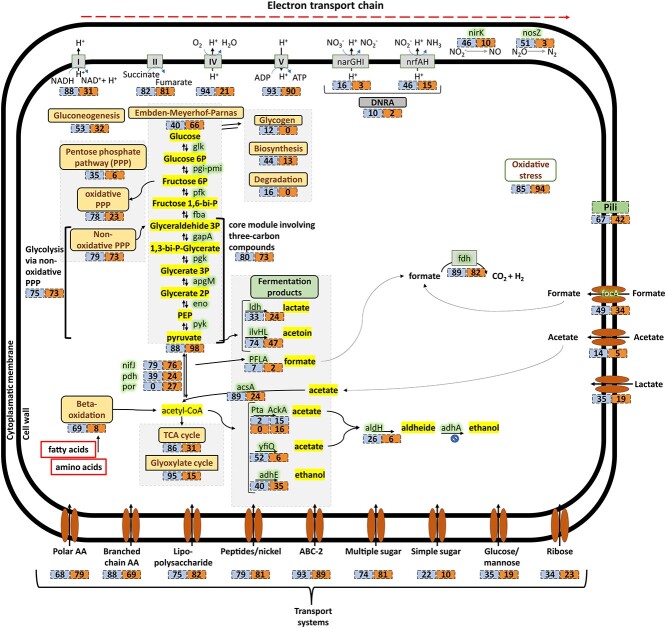
Metabolic model of the Chloroflexota MAGs_annot_ in activated sludge and methanogenic reactors. The percentage of MAGs_annot_ from AS and MET reactors appears in the boxes when the gene or metabolic pathway is present.

Aerobic and anaerobic uptake of different substrates is a shared trait exhibited by members of Chloroflexota [8, 9]. This widespread characteristic, regardless of the reactor type, was confirmed in all MAGs_annot_, as genes encoding for different transporters were identified (Fig. 5): ABC-2 type (93% MAGs_AS_, 90% MAGs_MET_), Peptide/nickel (79% MAGs_AS_, 81% MAGs_MET_), Lipopolysaccharide (75% MAGs_AS_, 82% MAGs_MET_), Multiple sugar (74% MAGs_AS_, 81% MAGs_MET_), Branched-chain amino acid (86% MAGs_AS_, 63% MAGs_MET_) and Polar amino acid (68% MAGs_AS_, 79% MAGs_MET_).

**Figure 6:**
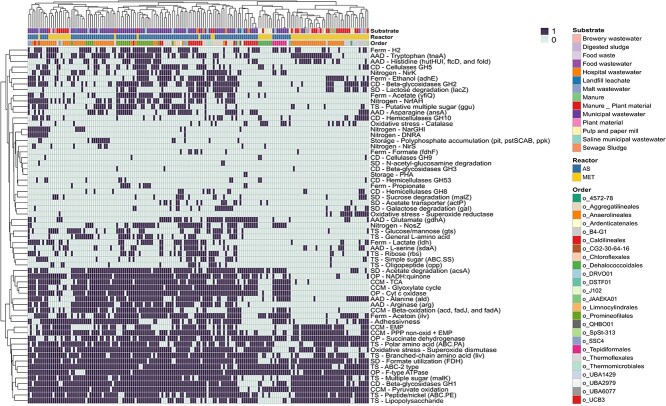
Hierarchical clustering (Euclidean distance) of the presence/absence of metabolic pathways in the MAGs_annot_. In the right part of the figure taxonomic assignment is shown at the order level.

Furthermore, other transporters, such as Putative multiple sugar (57% MAGs_AS,_ 29% MAGs_MET_), Glucose/mannose (50% MAGs_AS_, 25% MAGs_MET_), Ribose (49% MAGs_AS_, 25% MAGs_MET_), General L-amino acid (44% MAGs_AS_, 22% MAGs_MET_), Simple sugar (32% MAGs_AS_, 16% MAGs_MET_), Oligopeptide (23% MAGs_AS_, 11% MAGs_MET_), were more frequently identified in MAGs derived from activated sludge reactors. These findings indicated that the majority members of Chloroflexota could take up a wide range of sugars, fatty acids and amino acids as energy sources (Figs 5 and 6). This was demonstrated in previous experimental studies; wherein amino acid uptake was observed in both strictly anaerobic and facultatively aerobic Chloroflexota members [22, 30]. Genes encoding beta-oxidation, responsible for the degradation of fatty acids and branched-chain amino acids, were identified in more MAGs from AS reactors (69% MAGs_AS_, 8% MAGs_MET_), indicating a prevalent characteristic among them. While Chloroflexota species do exhibit a clear preference for simple sugars, complex polymers and amino acids, species capable of consuming short and long fatty acids have also been identified [8, 9]. Therefore, it might represent an important metabolic route for some Chloroflexota species to acquire carbon and reducing equivalents (Figs 5 and 6).

Glycolysis pathway (Embden–Meyerhof-Parnas) was complete in 40% MAGs_AS_ and 66% MAGs_MET_ (Figs 5 and 6, Supplementary Data 3). The remaining MAGs_annot_ could still achieve the glycolysis through a metabolic loop involving non-oxidative pentose phosphate pathway and the core module involving three-carbon compounds of glycolysis (75% MAGs_AS_ and 73% MAGs_MET_) (Figs 5 and 6) [92].

TCA cycle was found to be complete in 86% of MAGs_AS_ (31% MAGs_MET_) suggesting that terminal oxidation via the TCA serves as the primary energy source for members of Chloroflexota members in activated sludge systems (Figs 5 and 6). Additionally, the Glyoxylate cycle was identified in 31% of MAGs_AS_ (2% MAGs_MET_), suggesting the potential ability to utilize C2 compounds via the glyoxylate cycle for energy generation.

Furthermore, it was observed that 89% of MAGs_AS_ and 24% of MAGs_MET_ encoded acetyl coenzyme A synthetase (acsA), allowing for the conversion of acetate (a short-chain fatty acid) into acetyl-CoA. This finding is consistent with both isolated members and metatranscriptomic reports [33, 90]. This feature could be particularly advantageous in acetate-rich wastewater or in the absence of glucose [93]. It is worth noting that the acetate transporter gene (actP) was only present in 14% of MAGs_AS_ and 5% of MAGs_MET_, suggesting that these organisms may primarily rely on internal pools for the utilization of acetate as a carbon source [20]. Additionally, the majority of MAGs_annot_ (89% MAGs_AS_, 82% MAGs_MET_) were found to possess genes encoding formate dehydrogenase (fdh), which could potentially be used to reduce formate generated during anaerobic fermentation. This feature has been previously confirmed in some members of Chloroflexota [26, 33].

The ability to ferment is widely distributed in Chloroflexota. Most MAGs_annot_ encoded genes involved in producing at least one fermentation product, such as ethanol (aldH, 40% MAGs_AS_, 35% MAGs_MET_), lactate (ldh, 33% MAGs_AS_, 24% MAGs_MET_), acetoin (ivlHI, 74% MAGs_AS_, 47% MAGs_MET_), acetate (yfiQ, 52% MAGs_AS_, 6% MAGs_MET_) and hydrogen (yfiQ, 89% MAGs_AS_, 82% MAGs_MET_) (Fig. 6, Fig. S4, Supplementary Data 3). This was in accordance with previous reports showing that, in a medium supplemented with glucose and yeast extract, an isolate classified within the Anaerolineae class produced acetate, hydrogen, lactate, succinate, formate, propionate and/or ethanol as the main end products of fermentation [30–32, 94]. Hence, these features indicate that most of these Chloroflexota could perform fermentation.

### Chloroflexota from activated sludge systems have a facultative anaerobic metabolism

The majority of Chloroflexota members from activated sludge reactors exhibited a complete oxidative phosphorylation chain, including NADH:quinone oxidoreductase, succinate dehydrogenase, cytochrome c oxidase and/or F-type ATPase (94% MAGs_AS_). In contrast, only 2% of MAGs_MET_ showed this feature (Figs 5 and 6). These findings align with Anaerolineae members isolated from methanogenic reactors, which are typically described as obligate anaerobes [30–32, 90, 94]. However, it's worth noting that some Anaerolineae genomes have been found to contain genes for aerobic respiration [8, 9, 21, 95, 96]. It has been hypothesized that these complexes for aerobic respiration were likely acquired via horizontal gene transfer in some Chloroflexota members [97].

Considering that most of the Chloroflexota genomes detected in activated sludge reactors do not have isolated representatives in pure culture, the discovery of the first species capable of utilizing O_2_ within the Anaerolineae class only occurred after the in situ characterization of Ca. Villigracilis [9]. A recent study using in situ characterization indicated that members of Chloroflexota retrieved from activated sludge reactors can utilize oxygen, N_2_O and NO_2_^−^ [20]. Our findings suggest that most of the Chloroflexota members in activated sludge reactors are capable of using O_2_ as the final electron acceptor. Anoxic zones are likely common within large and denser flocs in activated sludge reactors due to limitations in oxygen diffusion [98]. The ability to ferment, found in most of the MAGs_AS_, allows Chloroflexota to survive in these anoxic microniches. Moreover, enzymes related to protection against oxygen and/or reactive oxygen species, such as superoxide reductase/desulfoferrodoxin, superoxide dismutase, and catalase, were widely distributed in Chloroflexota phylum (present in 85% of AS MAGs_annot_ and 95% of MET MAGs_annot_) (Fig. 6, Supplementary Data 3). These findings suggest that they are well prepared for defense against reactive oxygen species.

The majority of MAGs_AS_ have the potential to carry out the reduction of at least one nitrogen species (92% MAGs_AS_, 24% MAGs_MET_). This includes nitrate, with potential dissimilatory nitrate reduction to nitrite (narGHI, 16% MAGs_AS_, 3% MAGs_MET_), and the potential for dissimilatory nitrite reduction to ammonia (nrfAH, 46% MAGs_AS_, 15% MAGs_MET_). DNRA was present in 10% MAGs_AS_ and 2% MAGs_MET_. Genes for nitrite reduction to nitric oxide (nirK) were present in 46% MAGs_AS_ and 10% MAGs_MET_. Interestingly, 51% of the MAGs_AS_ (3% MAGs_MET_) contains a periplasmic nitrous oxide reductase (NosZ), indicating that nitrous oxide may also serve as a terminal electron acceptor. Therefore, Chloroflexota members could play a significant role in nitrite reduction and/or partial denitrification in activated sludge reactors (nirK, nosZ), suggesting a potential role in nitrogen removal from wastewater. The presence and use of genes related to the reduction of nitrogen species was reported by several studies [7, 20, 26, 33].

The ability to take up substrates under anoxic conditions as well as in presence of nitrate/nitrite was confirmed in members of Chloroflexota from activated sludge reactors, suggesting their character as facultative anaerobic chemoorganotrophs [9].

### Why are members of Chloroflexota so successful in WWTPs?

The most widely accepted hypothesis is that members of the Chloroflexota phylum are notoriously difficult to cultivate due to slow growth, particularly those belonging to the Anaerolineae class [23, 24]. Consequently, they are easily outcompeted by fast-growing heterotrophic anaerobes. The slow growth of Chloroflexota members has also been demonstrated by the replication index of Chloroflexota MAGs across multiple samples from anaerobic digesters [99]. In this context, Chloroflexota was found to be within the 90% of the total MAGs with a dereplication index between 1.1 and 2, indicating slow growth. Only 10% of the total community had values between 2 and approximately 4, which can be considered “fast growing”. On the other hand, isolated Anaerolineae members from methanogenic reactors require associations with other microbes (e.g. Archaea *Methanosaeta* spp.) for efficient growth [23].

Despite extensive efforts, only 14 species within Anaerolineae class have been successfully isolated from various environments, including anaerobic digesters, rice paddy soils, terrestrial aquifers, hydrothermal vents, and subseafloor sediment [30–32, 90, 94, 100–102]. The difficulty in isolating members of the Chloroflexota phylum in pure culture could also be due to the absence of key genes involved in B-vitamin biosynthesis are missing, as indicated by our results in most of the Chloroflexota MAGs (Supplementary Data 3). For instance, genes for thiamin (vitamin B1) biosynthesis (thiamine-phosphate synthase and thiamine- monophosphate kinase), biotin (vitamin B7) biosynthesis (adenosylmethionine-8-amino-7-oxononanoate ami-notransferase and biotin synthase) and adenosylcobalamin (vitamin B12) biosynthesis (cobalamin synthase and adenosylcobinamide-phosphate synthase) were absent as previously reported [21, 33]. This suggests that other microorganisms may support B-vitamin requirements for Chloroflexota community. Thus, the high abundances of Chloroflexota members may suggest that their ecophysiology provides them with some competitive advantage over other bacterial populations, but they likely require vitamins and cofactors supplied by other microorganisms.

In our study, we demonstrate that all MAGs have a potential versatile metabolism related to the hydrolysis, fermentation and respiration of complex and simple organic compounds. This versatility enables these microorganisms to survive in the most diverse microbiomes, demonstrating their adaptability to different or changing conditions. On the other hand, it has been extensively proposed that Chloroflexota plays an important role in granule and floc formation. Evidence for this is their growth as filaments and the fact that some members showed cellular adhesiveness [89]. A complete set of genes for the pilus assembly (pilA, CpaB, CpaE, CpaF, TadB, TadC) which favor adhesiveness, was annotated for 67% MAGs_AS_, 42% MAGs_MET_ (Figs 5 and 6). As has been already noted, pili are often involved in facilitating adhesion and colonization in a wide variety of scenarios. Thus, these characteristics could represent a selective advantage for Chloroflexota evidenced by their high abundance in WWTPs.

## Conclusions

In this study, we successfully addressed the initial questions through a comprehensive analysis of 264 genomes recovered from full-scale reactors.

Is the taxonomic composition of Chloroflexota the same in activated sludge and methanogenic reactors? Our findings suggest that the Chloroflexota taxonomic composition differs between activated sludge and methanogenic reactors. The Anaerolineae class was predominant in both systems, but with specific families in each.

Does the metabolic potential of Chloroflexota differ between both systems? Which carbon compound degradation pathways do they have? Genomes from both reactor types exhibit the potential to degrade complex organic matter and ferment a wide range of substrates. Our results suggest that Chloroflexota species from MET reactors are strict fermenters, while species from AS reactors also possess genes for both aerobic and anaerobic respiration, potentially playing a crucial role in nitrogen removal.

Our study provides a robust analysis and contributes valuable insights into the diversity and metabolic potential of the Chloroflexota phylum within WWTPs. This compilation of genomes serves as a valuable resource for generating hypotheses in future studies and as a starting point for targeted cultivation of previously uncultivated Chloroflexota members.

However, it is important to note that due to missing metadata and limited statistical power, establishing significant associations between diversity or genes of Chloroflexota and reactor performance remains challenging. Therefore, well-designed studies that incorporate experimental approaches are necessary for a comprehensive understanding of Chloroflexota's impact on reactor functionality. Future research utilizing metagenomic and metatranscriptomic approaches will further validate genomic predictions, advancing our knowledge of Chloroflexota physiology and its influence on reactor responses.

## Supplementary Material

9_Supplementary_material-clean_revised_ycae050

10_Suppl_Data_1-REVISADO_ycae050

11_Suppl_Data_2_ycae050

12_Suppl_Data_3_ycae050

## Data Availability

The raw metagenome sequences and MAG_sdRep_ generated in the present work have been deposited in the NCBI database with BioProject accession number PRJNA1037517. Scripts used in this study are available at Figshare (https://doi.org/10.6084/m9.figshare.24480880.v). Metagenomes and MAGs from published papers can be downloaded using the accession number associated with each respective work.
