# Supplementary material for: Unveiling the hidden diversity and functional role of Chloroflexota in full-scale wastewater treatment plants through genome-centric analyses
Source: ISME Commun. 2024 Apr 11;4(1):ycae050. doi: 10.1093/ismeco/ycae050 (PMC11653643; doi:10.1093/ismeco/ycae050)
Supplement: 9_Supplementary_material-clean_revised_ycae050 [file 9_supplementary_material-clean_revised_ycae050.docx]

Supplementary information:

Unveiling the hidden diversity and functional role of Chloroflexota in full-scale wastewater treatment plants through Genome-centric analyses

Patricia Bovio-Winkler^1^ • Angela Cabezas^2^ • Claudia Etchebehere^1^

Table S1. Basic statistics of shotgun sequence statistics (number of reads, read length, dataset size) of all metagenomes used for assembly in this study

Table 2. Statistics summary of the 16 species of Chloroflexota which met the criteria for HQ draft genomes and lack any closed representative species in the phylogenetic tree recovered in this study (Fig. 3 clusters A-O).

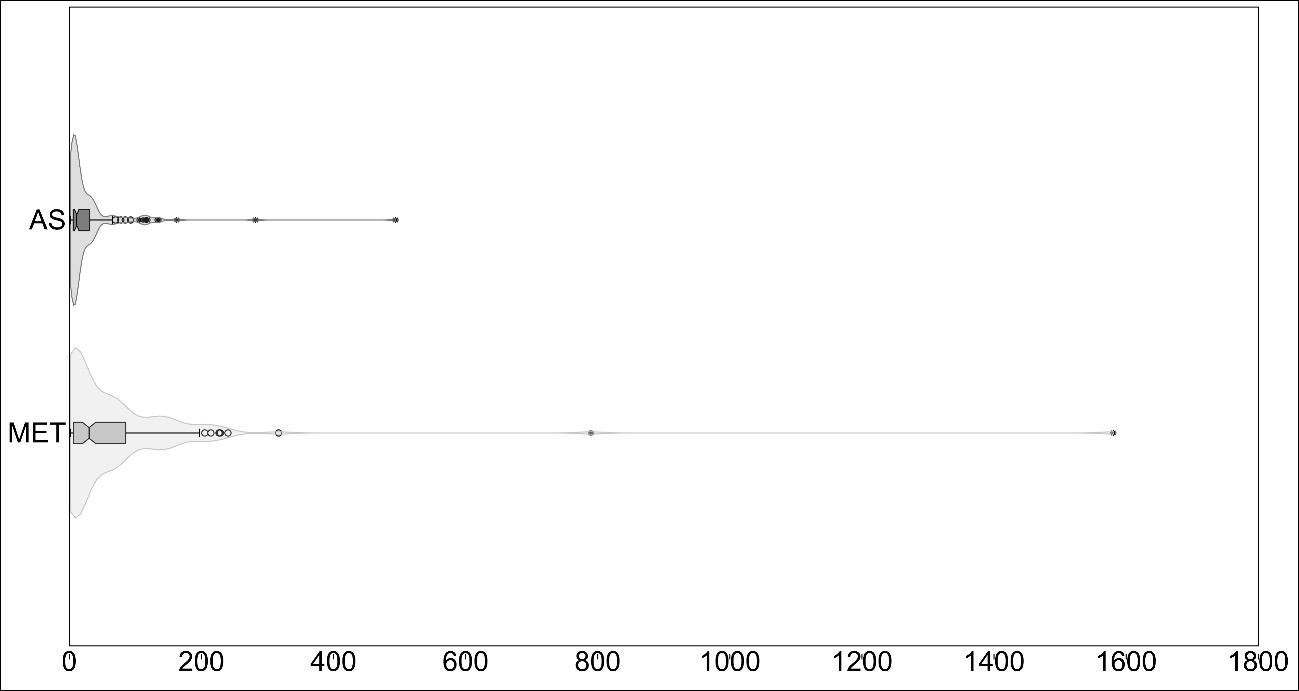


Figure S1. Violin plots showing the comparison of the genome coverage between MAGsQS from AS and MET reactors (normal distribution Shapiro-Wilk p-value = 0.00049, Mann-Whitney U test (Bonferroni corrected) p-value = 0.0004924)


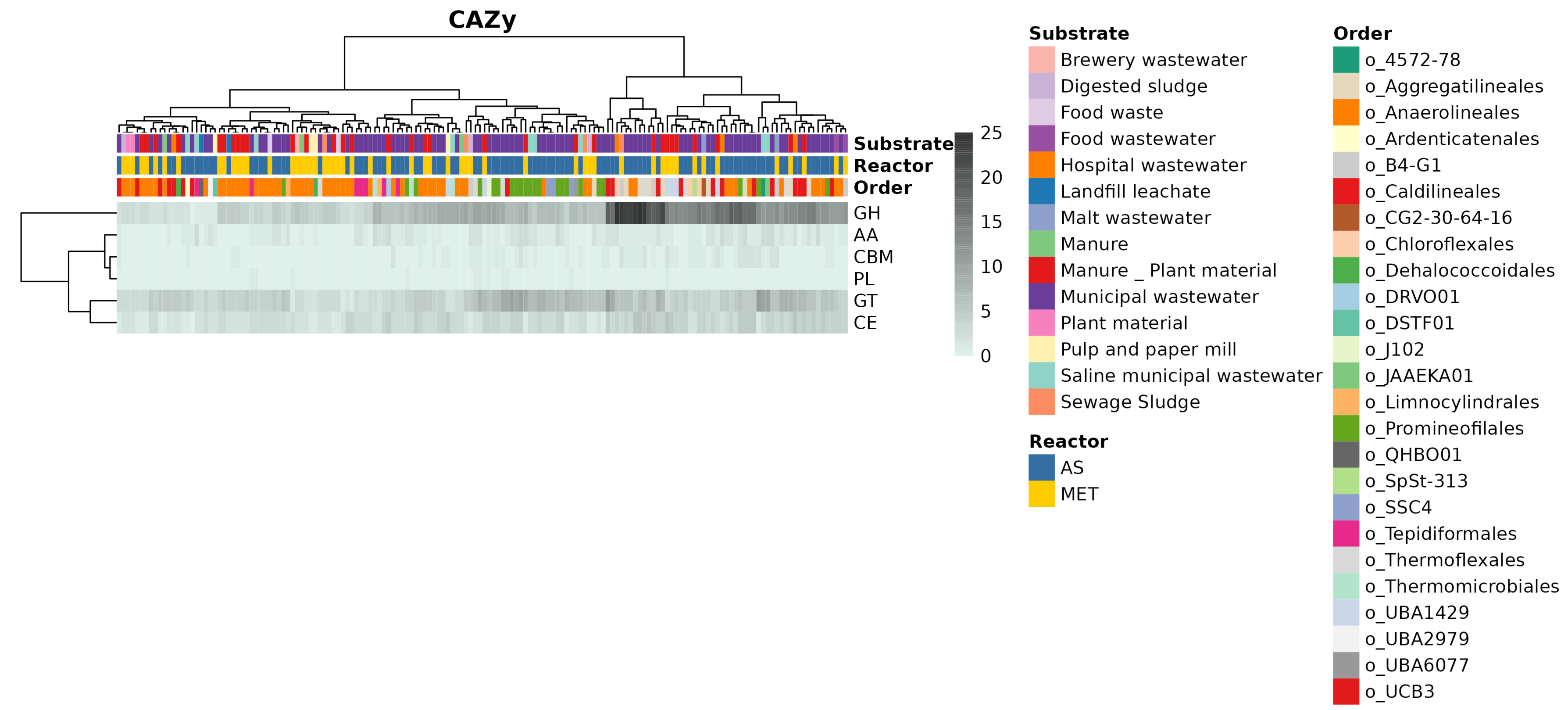


Figure S2. Heatmap showing the amount of CAZy classes within the Glycoside Hydrolase (GH), Glycosyl Transferase (GT), Carbohydrate Esterase (CE), Auxiliary Activities (AA), Carbohydrate Binding Module (CBM), and Polysaccharide Lyase (PL) families.


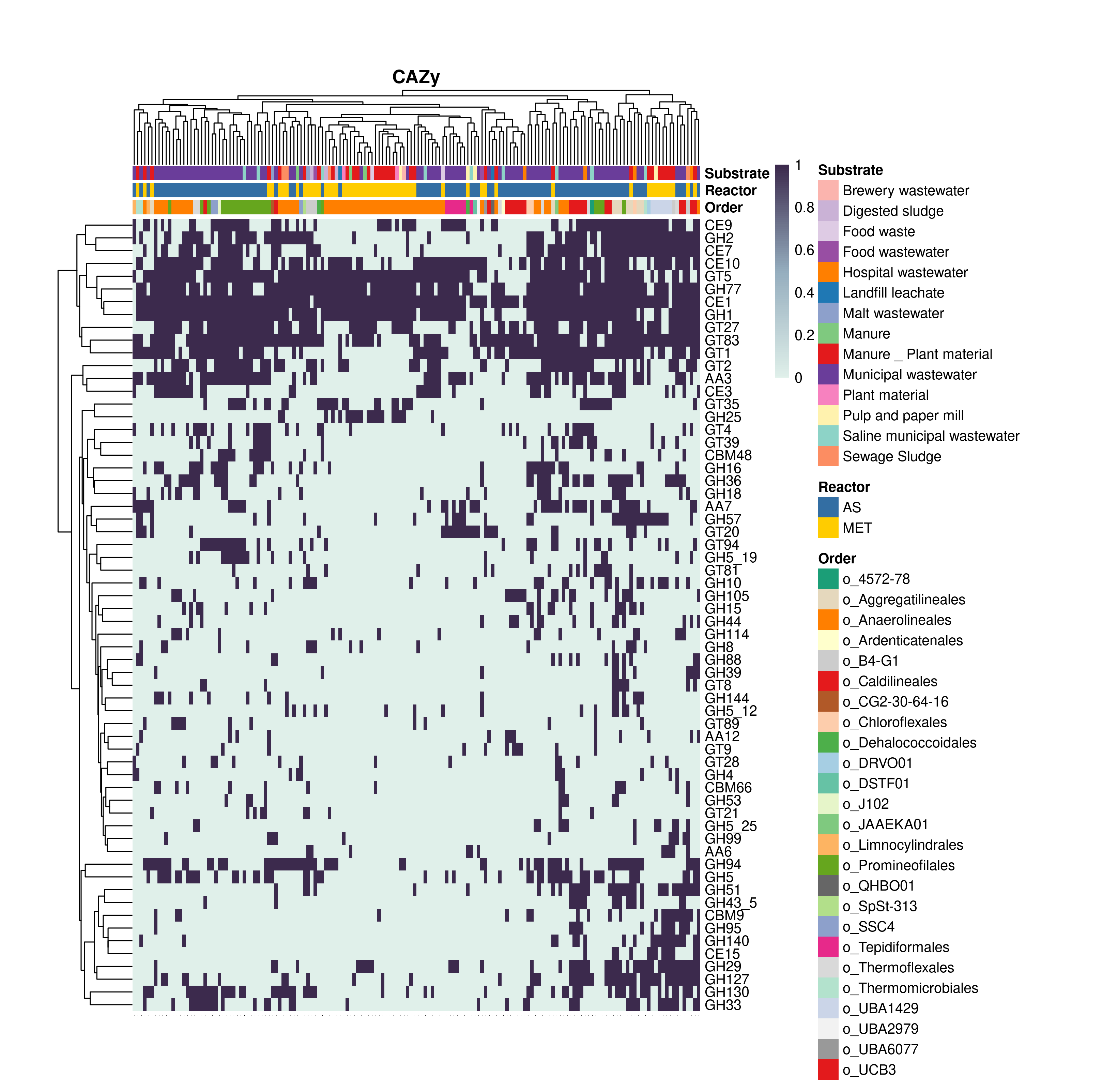


Figure S3. Hierarchical clustering (Euclidean distance) of the presence/absence of CAZy families identified in the MAGs_anot_. In the right part of the figure taxonomic assignment is shown at the order level.


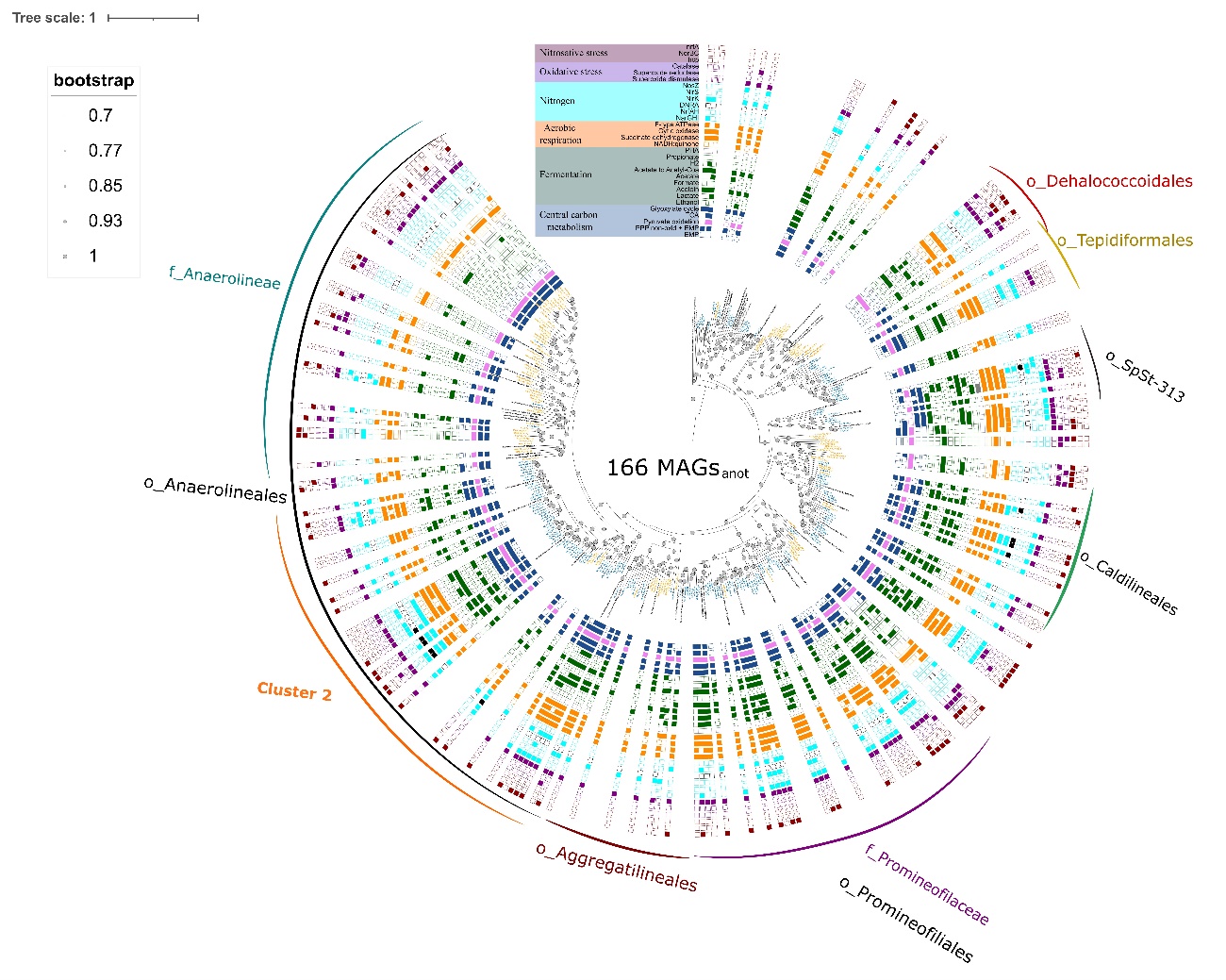


Figure S4. Phylogenomic tree of Chloroflexota and a heatmap displaying functions of interest for each Chloroflexota MAGs_annot_. The names of the MAGs were colored according to the type of WWTPs: blue for AS and orange for MET reactors. Sequences from the phylum Thermotogota were used as outgroup to root the tree.
